# Effects of robotic priming of bilateral arm training, mirror therapy, and impairment-oriented training on sensorimotor and daily functions in patients with chronic stroke: study protocol of a single-blind, randomized controlled trial

**DOI:** 10.1186/s13063-022-06498-0

**Published:** 2022-07-15

**Authors:** Yi-chen Lee, Yi-chun Li, Keh-chung Lin, Grace Yao, Ya-ju Chang, Ya-yun Lee, Chien-ting Liu, Wan-ling Hsu, Yi-hsuan Wu, Ho-ta Chu, Ting-xuan Liu, Yi-ping Yeh, Chieh Chang

**Affiliations:** 1 School of Occupational Therapy, College of Medicine, National Taiwan University, 17, F4, Xu Zhou Road, Taipei, Taiwan; 2 Division of Occupational Therapy, Department of Physical Medicine and Rehabilitation, National Taiwan University Hospital, Taipei, Taiwan; 3 Department of Psychology, National Taiwan University, Taipei, Taiwan; 4 Neuroscience Research Center, Chang Gung Memorial Hospital, Taoyuan, Taiwan; 5 School of Physical Therapy and Graduate Institute of Rehabilitation Science, College of Medicine, Chang Gung University, Taoyuan, Taiwan; 6 Healthy Aging Research Center, Chang Gung University, Taoyuan, Taiwan; 7 School and Graduate Institute of Physical Therapy, College of Medicine, National Taiwan University, Taipei, Taiwan; 8 Department of Rehabilitation, Taipei Tzu Chi Hospital, the Buddhist Tzu-Chi Medical Foundation, Taipei, Taiwan; 9 Rehabilitation Department, Taipei Hospital, Ministry of Health and Welfare, New Taipei City, Taiwan

**Keywords:** Stroke, Upper extremity rehabilitation, Bilateral motor priming, Bilateral arm training, Mirror therapy, Impairment-oriented training, Randomized controlled trial

## Abstract

**Background:**

Combining robotic therapy (RT) with task-oriented therapy is an emerging strategy to facilitate motor relearning in stroke rehabilitation. This study protocol will compare novel rehabilitation regimens that use bilateral RT as a priming technique to augment two task-oriented therapies: mirror therapy (MT) and bilateral arm training (BAT) with a control intervention: RT combined with impairment-oriented training (IOT).

**Methods:**

This single-blind, randomized, comparative efficacy study will involve 96 participants with chronic stroke. Participants will be randomized into bilateral RT+MT, bilateral RT+BAT, and bilateral RT+IOT groups and receive 18 intervention sessions (90 min/day, 3 d/week for 6 weeks). The outcomes will include the Fugl-Meyer Assessment, Stroke Impact Scale version 3.0, Medical Research Council scale, Revised Nottingham Sensory Assessment, ABILHAND Questionnaire, and accelerometer and will be assessed at baseline, after treatment, and at the 3-month follow-up. Analysis of covariance and the chi-square automatic interaction detector method will be used to examine the comparative efficacy and predictors of outcome, respectively, after bilateral RT+MT, bilateral RT+BAT, and bilateral RT+IOT.

**Discussion:**

The findings are expected to contribute to the research and development of robotic devices, to update the evidence-based protocols in postacute stroke care programs, and to investigate the use of accelerometers for monitoring activity level in real-life situations, which may in turn promote home-based practice by the patients and their caregivers. Directions for further studies and empirical implications for clinical practice will be further discussed in upper-extremity rehabilitation after stroke.

**Trial registration:**

This trial was registered December 12, 2018, at www.clinicaltrials.gov (NCT03773653).

## Background

Stroke rehabilitation has evolved in recent decades to the formulation of promising therapies that are effective, including robotic therapy (RT) [1], mirror therapy (MT) [2], bilateral arm training (BAT) [3], and, more recently, hybrid therapy [4] that may involve concurrent or sequential combinations of monotherapies. Especially relevant for technology-based rehabilitation is the use of RT as a priming technique.

### Robotic therapy (RT)

RT features intensive, repetitive, and task-specific practice that incorporates crucial components of motor learning and neurorehabilitation. Several systematic reviews indicate that RT leads to improvements in upper-extremity (UE) motor strength, motor impairment, and motor function in stroke patients [5, 6]. However, the evidence for positive effects in participation in daily activities remains uncertain [7], especially in patients with chronic stroke [6]. Current RT protocols should be further modified, such as combining RT with contemporary approaches (e.g., BAT or MT), to intensify the treatment and enhance the benefits on broader aspects of functional outcomes pertaining to daily activities [8].

### Mirror therapy (MT)

MT is a rehabilitation therapy in which a mirror is placed between the arms so that the mirror box blocks the vision of the paretic arm, and the individual can only see the actual movements of the nonparetic arm and its mirror reflection. At the same time, the participant is encouraged to move the paretic arm along with the mirror reflection. Our published studies have demonstrated that MT could induce greater improvements in motor functions (e.g., decreased motor impairment), movement control strategies (e.g., reduced shoulder abduction during forward reaching), and daily activities (e.g., improved functional independency) compared with conventional occupational therapy [9, 10]. MT also ameliorated sensory deficits and reduced pain, which may be attributed to the referral of sensation and increased cortical somatosensory representations [11]. In addition to MT treatment alone, growing literature suggests that combining MT with an adjuvant therapy such as RT can further intensify the treatment effect [9].

### Bilateral arm therapy (BAT)

BAT involves simultaneous practice of the same activities with both UEs in a symmetrical or alternating pattern and has been used as an effective UE rehabilitation intervention for individuals with stroke at all levels of severity [12]. Bilateral symmetrical movements allow for activation of the undamaged hemisphere and promotion of neural plasticity to facilitate movement control of the affected extremity [13]. BAT has been shown to reduce motor impairment, increase grip strength, and improve motor control (e.g., increase temporal and spatial efficiency and decrease online error correction during reaching tasks) [3]. Recent systematic reviews indicate that the evidence is insufficient to conclude that BAT has a definitive effect compared with other treatments [14] and recommend more research with adequate experimental, dose-matched designs, and sufficient statistical power [15].

### Chi-square automatic interaction detector to identify predictors of treatment success

Compared with traditional statistical analysis, chi-square automatic interaction detector analysis can provide more robust information and uncover patterns that are usually undetected by traditional statistical methods [16]. Furthermore, chi-square automatic interaction detector analysis can generate a decision tree that splits participants into subgroups with similar characteristics based on the identified predictor variables. This type of decision tree analysis can not only classify responders but can also identify predictive variables that may be relevant to treatment success.

### Movement-based robotic priming

Motor priming in neurorehabilitation can be defined as a change in behavior on the basis of previous stimuli and is an emerging strategy to facilitate motor relearning [4]. As a therapeutic possibility, bilateral RT may be used as a bilateral priming technique in combination with different task-oriented therapies [17], such as MT and BAT, and may yield differential benefits. This project aims to investigate the comparative efficacy of these different combinatory approaches based on the tenet of bilateral movement practice approach. The primary goal of the comparative efficacy trial will be to explore the differential effects of the hybrid intervention of RT combined with MT and BAT on motor function, daily function, sensory, mobility, life quality, self-efficacy, and motor control strategy in stroke patients. The retention effects and possible delayed response of the interventions will be examined at 3-month follow-up. The second aim of this study will be to identify predictors of changes in quality of life after the interventions using the Chi-square Automatic Interaction Detector (CHAID) method

## Methods and design

### Participants

Patients with stroke who meet the following criteria will be recruited: (1) ≥ 3 months’ onset from a first-ever unilateral stroke, (2) age range from 18 to 80 years, (3) baseline UE motor score on the Fugl-Meyer Assessment (FMA) > 10 [18], (4) no severe spasticity in any joints of the affected arm (Modified Ashworth Scale ≤ 3) [19], (5) able to follow study instructions (Mini-Mental State Examination Score ≥ 24) [20], (6) no serious vision deficits or other neurologic or major orthopedic diseases, (7) able to participate in a rehabilitation intervention program for 6 weeks, and (8) no participation in other studies during the study period and willing to provide written informed consent. Exclusion criteria are (1) acute inflammation, (2) major medical problems or poor physical condition that might interfere with participation, and (3) simultaneously participating in other medication or rehabilitation studies during the trial. However, the patients may continue relevant concomitant care and interventions such as standard physical therapy or taking needed medications.

### Sample size estimation

No published research to date has compared the effects of bilateral RT+MT, RT+BAT, and RT+impairment-oriented training (IOT). Thus, the sample size required for this project was calculated and estimated based on our published studies [8, 21, 22].

In a previous study, we randomized 21 stroke patients to two treatments: RT+task-oriented training and RT+IOT [21]. Between-group analyses revealed small to large effects on the FMA (*p* = 0.01, *η*^2^ = 0.43), Stroke Impact Scale (*p* = 0.046, *η*^2^ = 0.29), and Medical Research Council scale (*p* = 0.98, *η*^2^ < 0.01), respectively.

An additional 21 stroke patients were randomized to receive RT+BAT or RT [8]. Between-group analyses revealed small to large effects on the FMA (*p* = 0.82, *η*^2^ = 0.003) and Goal Attainment Scale total scores (*p* > 0.01, *η*^2^ = 0.633). We randomized 23 stroke patients to two treatment groups: MT and BAT [22]. Between-group analyses revealed small to large effects on the FMA (*p* = 0.67, *η*^2^ = 0.009) and Stroke Impact Scale Version 3.0 total scores (*p* = 0.02, *η*^2^ = 0.165), respectively.

In addition, we used the pilot data of three groups for the FMA proximal UE score (*p* = 0.051, *η*^2^ = .114) to calculate sample size. In light of our previous findings [8, 21, 22], we estimate that to achieve a statistical power of 0.80 with a two-sided type I error of 0.05 and with an estimated dropout rate of 10% to 15% at the 3-month follow-up, a total sample size of 96 with 32 subjects for each group will be sufficient.

### Design and procedure

This study is a single-blind, randomized controlled trial with pretest, posttest, and 3-month follow-up assessments. This comparative efficacy study will be based on a controlled trial with 3 arms: bilateral RT+MT, bilateral RT+BAT, and the control intervention of bilateral RT+IOT. The randomization sequence will be created by using a random number table with an allocation ratio of 1:1:1. Participants will be recruited from four study sites (i.e., National Taiwan University Hospital in Taipei, Chang Gung Memorial Hospital in Linkou, Taipei Hospital in New Taipei City, and Taipei Tzuchi Hospital in New Taipei City). Our therapists will screen eligible participants every day at each study site. When new participants are registered, a blinded research assistant will prepare a sheet containing the assigned group of the participants in a sealed envelope and give it to the therapist. A total of 96 eligible subjects will be stratified into four strata according to the side of lesion (right-side vs. left-side cerebrovascular accident) and the level of motor impairment (the cutoff will be 33 points on the FMA- UE Subscale) [23] at each of the four study sites to balance randomization assignment (4 strata with 24 subjects at each site). However, unblinding of participant’s group assignment is permissible when knowledge of the actual treatment is absolutely essential for further management of the patient.

The Ethical Committees for Human Research at the hospitals where participants are recruited have approved the study protocol. A well-trained and certified occupational therapist, who is blinded to the group assignment, study hypotheses, and intervention of the patients, will administer baseline, postintervention, and 3-month follow-up assessments. The baseline assessment will take place within 1 week before the start of the intervention, and the postintervention assessment within 1 week after the end of the intervention. Table 1 presents the timing of all study procedures.

**Table 1:**
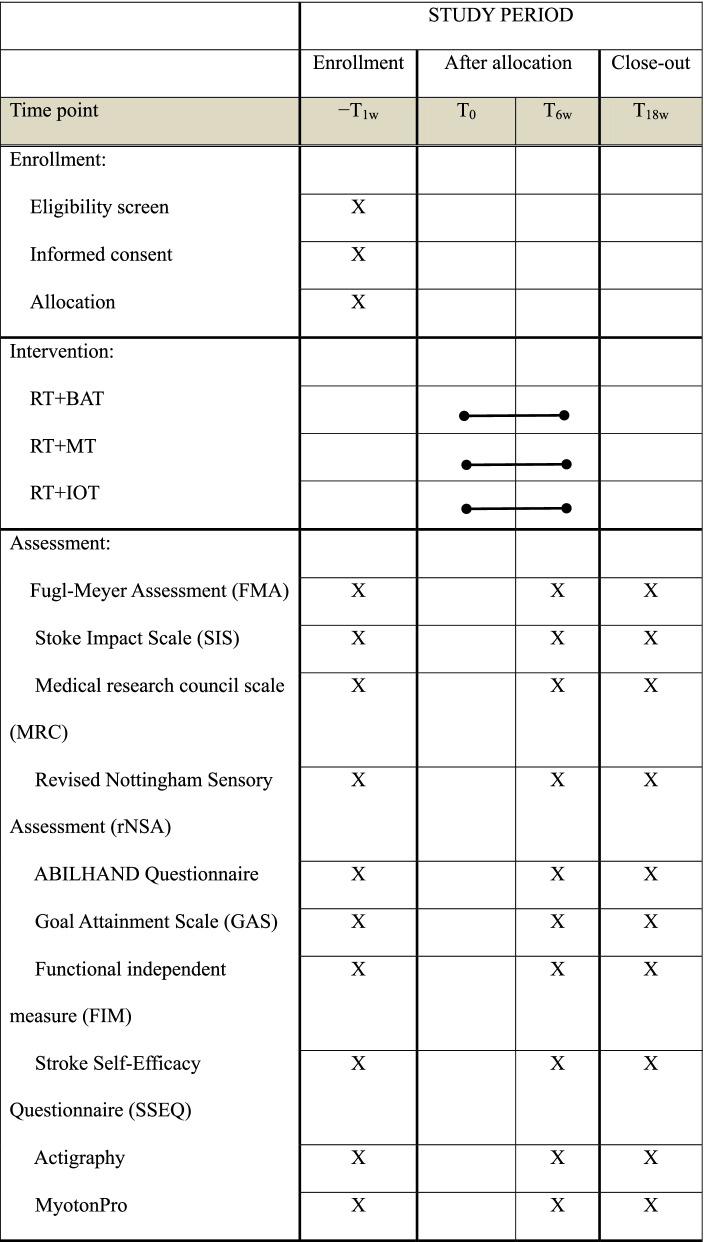
The schedule of enrolment, interventions, and assessments of this study protocol

### Intervention

Each treatment session will be delivered one-on-one by licensed occupational therapists. The Bi-Manu-Track robot (Reha-Stim Co., Berlin, Germany) will be used for the RT for 40 to 45 min in the three groups. Participants will sit at a height-adjustable table and practice 2 mirror-like movements: forearm pronation-supination and wrist flexion-extension with three computer-controlled modes: (1) passive-passive, with both arms being moved by the machine; (2) active-passive, with the unaffected arm driving the affected arm in a mirror-like fashion; and (3) active-active, with both arms actively moving against resistance. The speed of movement, the amount of resistance, and the range of movement can be adjusted individually on the Bi-Manu-Track. Computer games will be used to provide visual feedbacks to facilitate participation and motivation during the RT session. Participants are expected to perform approximately 1200 to 1600 repetitions of movements as bilateral robotic priming.

#### Experimental group 1: Bilateral robotic priming to be combined with bilateral arm training (RT+BAT)

After the robotic priming, participants will receive another 40 to 45 min of training in tasks focusing on bilateral symmetric movements of both UEs. The therapist will encourage the participants to perform the task with their paretic UEs as symmetrically as possible. The activities performed by the participant may involve 5-min intransitive movements (e.g., elbow flexion/extension or forearm pronation/supination) and 35 to 40 min of transitive tasks (e.g., flipping cards, scooping soup out of a bowl, or wiping the table), depending on the level of UE function. The tasks will be selected and graded on an individual basis to fit into the participants’ personal need for functional recovery.

#### Experimental group 2: Bilateral robotic priming to be combined with mirror therapy (RT+MT)

After bilateral robotic priming, participants will receive 40 to 45 min of MT. During MT, a wooden mirror box (41 × 50 × 33 cm^3^) will be placed in front of the participant to block the participant’s view of the paretic UE performance. The participants will be asked to use their less paretic UE to perform certain functional tasks and to observe the less paretic UE movements reflected from the mirror. Simultaneously, the therapist will encourage the participant to perform the task as symmetrically as possible with the paretic UE behind the mirror. The MT protocol will be similar to that of BAT except for the use of mirror feedback in MT. The activities and tasks performed by the participant will depend on the level of UE function and the participant’s personal need for recovery.

#### Control intervention group: Bilateral robotic priming to be combined with impairment-oriented training (RT+IOT)

IOT is an approach developed to restore body functions after stroke, including remediation of motor impairments, rather than restoring functional performance [24]. Since motor impairments are prevalent in patients with stroke, IOT is a common approach in stroke rehabilitation. Therefore, we chose RT+IOT as the control intervention group in this study.

After bilateral robotic priming, participants will receive 40 to 45 min of IOT. Participants in the control group will receive systematic repetitive training, the Arm Basis Training or the Arm Ability Training, according to each participant’s impairment level. Participants with a baseline FMA-UE score lower than 35 or without the ability of a precision grip will receive the Arm Basis Training, whereas participants with a baseline FMA-UE score of 35 or more and the ability of a precision grip will receive the Arm Ability Training.

The Arm Basis Training protocol will focus on the capacity of selective movements in all arm segments (shoulder, elbow, forearm, wrist, and fingers), from isolated movements without weight bearing of the affected UE to the movements against and with gravity and from single-joint motions to multiple-joint motions. The Arm Ability Training targets different sensorimotor abilities, such as speed, grasping, aiming, dexterity, tracking, steadiness, accuracy, and endurance with eight training activities (aiming, tapping, cancelation, turning coins, maze tracking, bolt and nut, placing small objects, and placing large objects) [21, 24, 25]. Table 2 summarizes the treatment regimen for the experimental and control groups.

**Table 2:** Treatment regimen for the experimental and control groups

Group	Treatment	Duration	Frequency	Intensity
Experimental 1	Bilateral RT+BAT	90 min/day 45 min: RT 45 min: BAT	3 days/week	6 weeks
Experimental 2	Bilateral RT+MT	90 min/day 45 min: RT 45 min: MT	3 days/week	6 weeks
Control	Bilateral RT+IOT	90 min/day 45 min: RT 45 min: IOT	3 days/week	6 weeks

*RT* Robotic therapy, *BAT* Bilateral arm training, *MT* Mirror therapy, *IOT* Impairment-oriented training

### Outcome measures

#### Primary outcome measurement

##### Fugl-Meyer Assessment (FMA)

The UE subscale of the FMA will be used to assess motor impairment [18]. There are 33 UE items measuring the movements and reflexes of the shoulder/elbow/forearm, wrist, hand, and coordination/speed. Each score is on a 3-point ordinal scale (0 = cannot perform, 1 = performs partially, 2 = performs fully), with a maximum score of 66 as optimal recovery. The subscale score of a proximal shoulder/elbow (FMA s/e: 0–42) and a distal hand/wrist (FMA h/w: 0–24) will be calculated to investigate the treatment effects on separate UE elements. The FMA has good reliability, validity, and responsiveness in stroke patients [26].

#### Secondary outcome measurements

##### Stroke Impact Scale Version 3.0

The Stroke Impact Scale Version 3.0 is a stroke-specific health-related quality of life instrument (Duncan et al., 2003). It consists of 59 items assessing eight domains: strength, hand function, activities of daily living/instrumental activities of daily living, mobility, communication, emotion, memory, thinking, and participation. Items are rated on a 5-point Likert scale, with lower scores indicating greater difficulty in task completion during the past week. The Stroke Impact Scale Version 3.0 has satisfactory reliability, validity, and responsiveness in stroke patients [27].

##### Medical Research Council scale

The Medical Research Council scale will be used to measure muscle strength of UE joints of the affected arm by the 6-point ordinal scale (0 = plegic, 5 = resisted to maximal strength, full power compared with the unaffected side), and the average Medical Research Council scale score will be calculated. The Medical Research Council scale demonstrates reliability in muscle power measurement [28].

##### Revised Nottingham Sensory Assessment

The Revised Nottingham Sensory Assessment will be used to evaluate changes in sensation, including tactile sensation, proprioception, and stereognosis of different segments of the body [29]. The Revised Nottingham Sensory Assessment is scored on a 3-point ordinal scale (0–2), with a lower score suggesting greater sensory impairment. The psychometric properties have been established for patients with stroke [29].

The *ABILHAND Questionnaire* is an inventory of 23 manual activities on which the patient provides his or her self-perceived performing difficulty on a 4-level scale (0 impossible, 1 great difficulty, 2 some difficulty, 3 easy) [30]. Its reliability and construct validity has been confirmed in stroke patients [30].

The *Goal Attainment Scale* is an individualized outcome measure of the achievement of each participant’s expectation in the course of the intervention. The goals will be set before the intervention and scored the day before the treatment (pretest), after 6 weeks of treatment (posttest), and follow-up.

The *Functional Independence Measure* is an 18-item measurement tool that assesses a patient’s self-care, sphincter control, transfer, locomotion, communication, and social cognition ability [31], with a higher score (maximum score, 126) indicating less disability. The Functional Independence Measure has good inter-rater reliability, construct validity, and discriminant validity [31].

The *Stroke Self-Efficacy Questionnaire* is a 13-item questionnaire that measures a participant’s confidence in functional performance from stroke, rated from 0 (no confidence at all) to 10 (complete confidence). A higher score (maximum score, 130) indicates higher self-efficacy in functional performance from stroke. The Stroke Self-Efficacy Questionnaire has high internal consistency and good criterion validity [32].

The *Actigraphy* is an ActiGraph GX3 accelerometer (ActiGraph, Shalimar, FL, USA) that is worn on the wrist and may record activity levels of the patient’s UE for 3 consecutive days before and after the intervention. The accelerometer will record the number of moves each minute, and the average counts of moves per minute will be calculated.

##### Myoton Pro

The functional state of skeletal muscle, that is, muscle tone, elasticity, and stiffness, will be objectively assessed by the Myoton Pro (Muomeetria Ltd, Tallinn, Estonia) device after interventions.

##### Adherence

The following strategies will be implemented to increase participants’ adherence and retention to this study. Transportation is provided to participants to and from home to the study sites. Therapists will make a phone call to each participant every week and visit the participants at home as need. Participant’s adherence will be measured based on session attended and completed.

##### Data management and quality

All documents in this study will be stored in a locked cabinet, including signed informed consent forms and data recording sheets. The electronic database will be protected by using a password. No identifiable personal information of the participants will be available from the electronic database. The principle investigator (Keh-chung Lin) was in charge of data monitoring in this study. Therefore, there was no additional data monitoring committee. The second author of this study (Yi-chun Li) will conduct interim data analyses and the principle investigator (Keh-chung Lin) will make the final decision to terminate the trial.

##### Adverse event monitoring and reporting

The vertical numerical rating scale supplemented with a faces rating scale [33] and a self-reported assessment will be provided to evaluate adverse effects on fatigue and pain severity, respectively. Both assessments, using 11-point scale (0 = no fatigue/pain to 10 = worst possible fatigue/pain), will be applied after each training session and at 3 months in the follow-up period. The therapist may adjust the practice activities based on the participant’s self-perceived burdens. The reliability and validity of the vertical numerical rating scale supplemented with a faces rating scale in measuring fatigue intensity in patients with stroke are supported by previous study [33]. In addition to pain and fatigue, other adverse events will be monitored and reported, including fall, seizure, dizziness, and hypertension.

### Statistical analyses

One-way analysis of variance and *χ*^2^ will be used to analyze differences in baseline characteristics and outcome measures among the three groups. Analysis of covariance will be conducted at the assessments, followed by a post hoc analysis using the Bonferroni test to identify the direction of the effects. Statistical significance will be set at 0.05 for all comparisons. We will use the “pretest” performance as a covariate to parcel out the possible confounding effects of differences in baseline performance. The group will be the independent variable and the posttest and follow-up test performance will be the dependent variable. The missing data will be adjusted based on the last observation carried forward (Herman, 2009). Partial eta squared (*η*^2^) will also be estimated to determine the group difference for each outcome measure. Predictors of intervention outcomes will be estimated with the chi-square automatic interaction detector method. Data analyses will be performed using the SPSS Statistics 18.0 software (IBM Corp, Armonk, NY).

## Discussion

The study goal is to investigate the comparative efficacy of RT+MT, RT+BAT, and RT+IOT after stroke. The outcome measures are selected based on our previous studies [8, 10, 21, 22] and are in line with the International Classification of Function framework to facilitate interpretability of the functional significance of treatment outcomes [34]. According to our previous research [8, 10, 21, 22], hybrid bilateral therapy (RT+MT and RT+BAT) may result in greater recovery of the primary outcome (i.e., FMA) compared with the control condition (RT+IOT). The RT+MT group may improve sensory function more than the RT+BAT and the control groups. In addition, the combined use of subjective and objective assessments would provide more comprehensive information regarding participants’ actual and perceived improvements.

Our hybrid regimens are unique in using bilateral robotic practice as a priming technique to augment bilateral task practice with and without mirror visual feedback. The anticipated benefits of robot-assisted bilateral movement priming in stroke rehabilitation may be associated with balanced excitability between the ipsilesional and contralesional hemispheres [35], positive reorganization in the motor cortex [36, 37], and improvements in later voluntary and complicated task performance [38]. The context-related and goal-directed practice in our treatment protocol may assist the patients to transfer therapeutic gains in clinics to their real-life environment and generalize motor improvements to performance of daily activities [21].

Mirror visual feedback of the unaffected UE is provided when both UEs perform bilateral tasks in the RT+MT group, which is expected to have more improvements in sensory function over the other two groups. It is possible that the illusory visual feedback to be substituted for inadequate proprioceptive input of the affected UE may activate multimodal neurons in parietal cortex [39]. This activation may contribute to improvements in somatosensory deficits [40, 41], which are frequent in patients recovering from stroke [42]. Given the promise of improving sensorimotor outcomes using mirror visual feedback during bilateral task practice, our research will study the therapeutic benefit of RT hybridized with MT relative to the dose-matched comparison treatment.

The findings of this protocol are expected to contribute to the research and development of technology-based stroke rehabilitation that will promote the use of robotic devices for streamlining stroke motor rehabilitation and the use of accelerometers for monitoring activity level in real-life situations. The activity monitors may be useful for providing behavioral feedback to the participants regarding their actual amount of movement. We anticipate that this research of the accelerometer will lead to increased use of self-monitoring in the home environment, which may in turn promote home-based practice by the patients and their caregivers. The findings can be applied to update the evidence-based protocols in the postacute stroke care program and to translate the evidence into practice and health care decision making.

### Protocol version

The 3^rd^ version

### Trial status

At the time of submission, the study is recruiting participants.

## Data Availability

Not applicable.

## Abbreviations

BAT: Bilateral arm training
FMA: Fugl-Meyer Assessment
IOT: Impairment-oriented training
MT: Mirror therapy
RT: Robotic training
UE: Upper extremity
